# Housing Insecurity and Threats of Utility Shut‐Offs Among Cancer Survivors in the United States, BRFSS 2022–2023

**DOI:** 10.1002/cam4.71436

**Published:** 2025-12-04

**Authors:** Tina Duong Nguyen, Jan M. Eberth, Elochukwu Ezenwankwo, Gabriel L. Schwartz

**Affiliations:** ^1^ Department of Health Management and Policy, Dornsife School of Public Health Drexel University Philadelphia Pennsylvania USA; ^2^ Sidney Kimmel Comprehensive Cancer Center Jefferson Health Philadelphia Pennsylvania USA; ^3^ Urban Health Collaborative, Dornsife School of Public Health Drexel University Philadelphia Pennsylvania USA

**Keywords:** BRFSS, cancer, cancer survivorship, housing, social determinants of health

## Abstract

**Background:**

The financial burden of cancer treatment can increase the risk of housing insecurity for patients undergoing treatment and survivors.

**Objective:**

To evaluate the burden of housing and utility insecurity among cancer survivors compared to individuals without a cancer history, examine outcome differences by housing tenure (renters vs. homeowners) and treatment status (active vs. posttreatment), and identify predictors of housing insecurity.

**Methods:**

We analyzed data from 14 states that completed the Social Determinants and Cancer Survivorship modules of the 2022 and 2023 Behavioral Risk Factor Surveillance System (BRFSS), yielding 5499 respondents with a previous cancer diagnosis (excluding skin cancers) and 61,883 respondents without a cancer diagnosis. We estimated prevalences and fit logistic regressions.

**Key Results:**

Cancer history was associated with greater odds of housing (AOR 1.43, 95% CI: 1.18–1.74) and utility (AOR 1.36, 95% CI: 1.09–1.69) insecurity, but this varied by treatment timing and housing tenure. Patients currently undergoing treatment were more likely to report housing and utility insecurity (AOR 1.96, 95% CI: 1.28–3.01 and AOR 1.67, 95% CI: 1.06–2.61, respectively) than individuals without a history of cancer. Such insecurity was elevated even after treatment for renters, but not for homeowners. In absolute terms, 34.7% of renters with a cancer history reported housing insecurity, compared to 7.1% of their homeowner counterparts.

**Conclusions:**

Cancer diagnosis and treatment can contribute to housing and utility insecurity during and after treatment. Addressing this through targeted interventions within both healthcare systems and social policy may mitigate hardship and improve well‐being.

## Graphical abstract

Using the latest BRFSS data, our study found that nearly 2 in 5 renters and more than 1 in 9 homeowners currently undergoing cancer treatment cannot pay their housing or utility bills. We also identified subgroups of cancer survivors at elevated risk of housing insecurity and threats of utility shut‐offs, highlighting the urgent need for multilevel interventions that support this vulnerable group of patients and survivors.

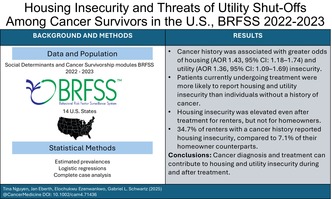

## 1 Introduction

Social determinants of health have significant impacts across the cancer continuum, from access to preventive care to treatment availability and survival outcomes [1, 2]. Cancer itself, however, can also give rise to adverse social determinants. Cancer diagnoses often result in significant financial hardship due to medical expenses, treatment‐related job loss, lost work hours, and increased caregiving needs [3, 4]. In 2019, the national patient economic burden totaled $21.1 billion [5]. As a result, 42% of cancer patients lose their entire life savings within 2 years of diagnosis [6]. This financial insolvency is more likely with more advanced disease progression, ongoing treatment, and various sociodemographic factors, including female gender, Medicaid enrollment, lack of insurance, lower income, and larger household size [6, 7]. Importantly, financial hardship can adversely affect cancer outcomes. Multiple cross‐sectional and cohort studies have linked financial toxicity to increased pain, higher symptom burden, lower health‐related quality of life, decreased satisfaction with care, and higher risk of mortality [7, 8, 9, 10].

Housing is particularly vulnerable to disruption by financial hardship throughout cancer treatment and survivorship. The financial toxicity of cancer care can force individuals to make trade‐offs between paying for treatment and maintaining stable housing [11, 12, 13]. Housing insecurity, characterized by the absence of safe, affordable, and stable housing, is linked to delays in medical care, lack of a usual source of care, postponement of medications, and difficulty recertifying eligibility for public insurance programs [14, 15, 16]. Individuals, especially renters and non‐homeowners, who are forced to relocate due to unaffordable housing are more likely to have unmet medical needs, worse general health, and greater psychological distress [17, 18]. Nor does housing insecurity risk end with treatment completion. Individuals with a cancer history, particularly those in younger age groups, are less likely to own homes and more likely to have negative net worth or carry debts [19]. This ongoing economic instability contributes to psychological distress, delayed or forgone medical care, and lower quality of life among survivors [20, 21].

To advance understanding of housing challenges among cancer survivors, our study addressed three critical questions. First, we estimated the prevalence of housing and utility insecurity among individuals currently undergoing cancer treatment and those who completed treatment. Second, we examined whether a history of cancer diagnosis was associated with an increased likelihood of experiencing housing and utility insecurity. Third, we investigated whether housing and utility insecurity differed between renters and homeowners, and whether these differences varied by key demographic and socioeconomic characteristics.

We considered these questions essential for addressing key gaps in the existing literature that currently limit the ability of governments, healthcare systems, and social service providers to formulate and implement effective policies and interventions. For instance, most studies examining housing insecurity among individuals with a history of cancer did not distinguish between those currently undergoing treatment and those who completed treatment [22, 23, 24]. This differentiation is critical for understanding short‐term and long‐term impacts of cancer diagnosis and treatment on financial stability and housing outcomes. Moreover, limited research has examined housing‐related financial hardship among cancer patients and survivors across diverse socioeconomic and demographic groups, particularly concerning home ownership status, that is, renters and homeowners.

Including both housing and utility insecurity in our analysis provided a more holistic understanding of material hardship experienced during and after cancer care. While housing insecurity captures instability related to shelter, such as unaffordable rent or risk of eviction, utility insecurity reflects the inability to afford or maintain basic utility services like electricity, water, heating, and cooling. These forms of insecurity often co‐occur and can compound one another, particularly among low‐income populations [25]. For individuals in cancer treatment or survivorship, utility insecurity can hinder health and recovery by limiting medication storage and safe indoor conditions, especially since certain cancer therapies can interfere with physiological thermal regulation [26]. Examining housing and utility insecurity together provides a better understanding of these intersecting challenges, enabling more targeted and effective policy strategies.

## 2 Methods

### 2.1 Data Source and Variables

We used BRFSS data from 14 states that completed the Social Determinants and Health Equity (SD/HE) and Types of Cancer/Cancer Treatment (TC/CT) modules in 2022 and 2023. These states were Connecticut, Delaware, Idaho, Mississippi, Missouri, Nevada, New Jersey, North Carolina, Ohio, Puerto Rico, Rhode Island, Utah, Vermont, and Wisconsin. Our analytic sample represents an estimated 28.8 million US adults, or about 10.8% of the national adult non‐institutionalized population. The data were pulled together and reweighted appropriately following the BRFSS recommendations [27].

#### 2.1.1 Independent Variables: Cancer History and Treatment

Our primary independent variables were a history of cancer diagnosis (yes/no) and current treatment status (current cancer treatment vs. completed treatment). We defined a history of cancer diagnosis as a “yes” response to the question, “Has a doctor ever told you you had melanoma or any other type of cancer?” We defined current treatment as a response “yes” to the question “Are you currently receiving treatment for cancer?” and completed treatment as a response “No, I've completed treatment” to the same question.

We excluded all skin cancers, both melanoma and nonmelanoma, based on responses to the question, “What kind of cancer is it?” We did so to ensure a comparable analysis across conditions, as treatment for many skin cancers is comparatively much less expensive, debilitating, and invasive than other cancer types. We also excluded respondents who refused treatment, those who had not started treatment, and those who reported that treatment was not necessary. For all variables, we excluded respondents with “don't know/not sure” or “refused to answer” responses.

#### 2.1.2 Outcomes: Housing and Utility Insecurity

Our primary outcomes were two indicators of housing and utility insecurity, specifically: (A) reported inability to pay mortgage, rent, or utility bills, and (B) experience of an electric, gas, oil, or water company threatening to shut off services. We defined housing insecurity as a response of “yes” to the question, “During the last 12 months, was there a time when you were not able to pay your mortgage, rent, or utility bills?” We defined utility insecurity as a response of “yes” to the question, “During the last 12 months, was there a time when an electric, gas, oil, or water company threatened to shut off services?” We excluded all missing responses.

#### 2.1.3 Covariates and Sociodemographic Predictors

We also examined sociodemographic factors as covariates, including sex (binary; male and female), age (categorical: 18–49, 50–64, and 65 and older), race/ethnicity (categorical: White, Non‐Hispanic; Black, Non‐Hispanic; Latino, Hispanic; American Indian/Alaskan Native, Non‐Hispanic; Asian, Non‐Hispanic, and Other Race, Non‐Hispanic), educational attainment (high school or less vs. at least some college or technical school), and rural/urban status.

**FIGURE 1:**
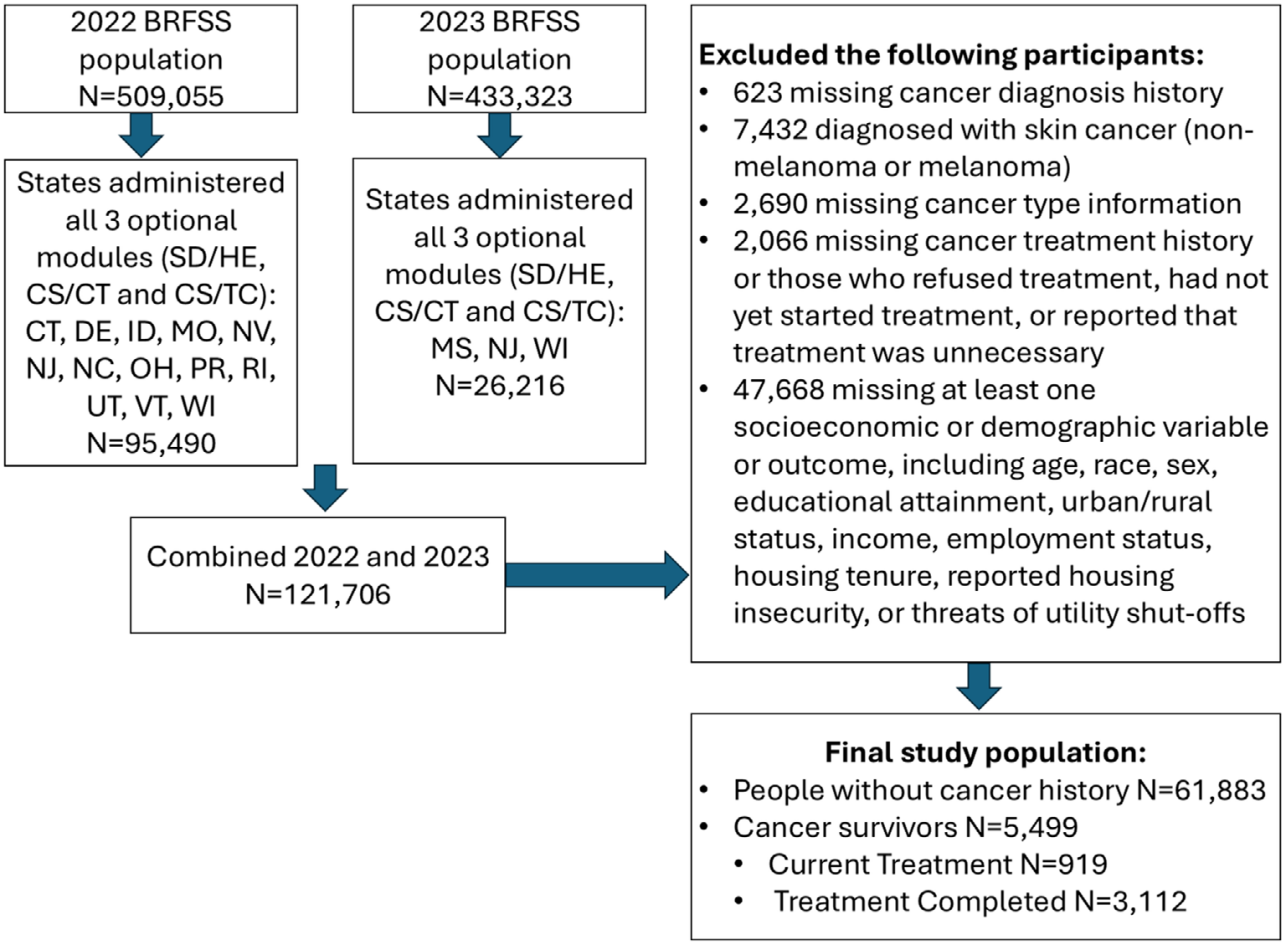
Flowchart of inclusion and exclusion criteria for the final study population.

Socioeconomic indicators such as income levels, employment, and housing tenure were examined as potential confounders and mediators. Income levels were recoded into three groups: less than $50,000, $50,000 to less than $100,000, and $100,000 or more. Employment status was recoded into three groups: employed (employed for wages and self‐employed), retired, and not working, including individuals who were out of work, homemakers, students, and unable to work. We excluded all respondents with “don't know/not sure,” “refused to answer,” or missing responses. The final study population and missing data are summarized in Figure 1.

### 2.2 Statistical Analysis

All analyses were conducted with SAS 9.4 software, accounted for the BRFSS's complex sampling design, and were weighted to be representative of participating states' adult, non‐institutionalized populations.

We first used descriptive statistics to analyze the demographic and socioeconomic characteristics of the sample. We then calculated the prevalences of housing and utility insecurity among individuals without a cancer history, those with a history of cancer diagnosis, those currently undergoing treatment, and those who completed treatment. The housing and utility insecurity prevalences were estimated both overall and stratified by housing tenure (renters vs. homeowners).

We next explored whether there were differences in the outcomes among individuals with a history of cancer diagnosis across different demographic and socioeconomic characteristics. We performed a series of design‐based logistic regressions to examine the relationship between various sociodemographic and economic factors and the outcomes. These factors, including sex, housing tenure, age, race/ethnicity, income, employment, and educational attainment, were selected based on prior literature identifying them as important determinants of housing insecurity and health. We conducted both unadjusted (bivariate) and adjusted (multivariate) logistic regression models. To account for multiple testing, statistical significance for the bivariate models was determined using false discovery rate (FDR)–adjusted *p* values (Benjamini–Hochberg correction). Confounder adjustment in the multivariate models was guided by directed acyclic graphs (DAGs) to ensure appropriate control for confounding while avoiding overadjustment for potential mediators. Specifically, models were adjusted as follows: for sex, adjusted for age and race; for age, adjusted for sex and race; for race, adjusted for sex and age; for education, adjusted for sex, age, and race; for housing tenure, adjusted for sex, age, race, education, income, and employment; for income, adjusted for sex, age, race, education, and employment; and for employment, adjusted for sex, age, race, and education.

Finally, we performed multivariable logistic regressions evaluating the relationship between having a history of cancer diagnosis (as well as current/completed cancer treatment status) and housing or utility insecurity. In both models, we adjusted for sociodemographic characteristics including age, sex, race/ethnicity, urban/rural status, and educational attainment, as guided by DAGs, as well as state and interview month fixed effects. The reference group consisted of individuals without a history of cancer. The analysis was conducted both overall and stratified by housing tenure (renters vs. homeowners). We also ran separate models controlling possible mediating paths through socioeconomic status, such as income and employment. We also conducted sensitivity analyses in which we included marital status and respondent‐reported long COVID as potential confounders [28, 29].

## 3 Results

### 3.1 Sample Descriptives

Sample characteristics and key study variables are summarized in Table 1. On average, those with a history of cancer were more likely to be female, older, homeowners, low‐income, and retired than those without a history of cancer. Notably, a higher proportion of individuals currently undergoing cancer treatment reported earning less than $50,000 a year, compared with those who completed cancer treatment (47.9% vs. 42.4%). Similar patterns were observed for employment: 23.7% of people currently undergoing treatment reported not working, compared to 13.2% among those who completed treatment. This was driven by both higher levels of employment and higher rates of retirement among those who completed cancer treatment. There were no significant differences in urban/rural status and educational attainment between groups.

**TABLE 1:** Sample characteristics^a^.

	No cancer (*N* = 61,883)		Ever cancer^b^ (*N* = 5499)		Current treatment^c^ (*N* = 919)		Treatment completed^c^ (*N* = 3112)	
	*N*	%	*N*	%	*N*	%	*N*	%
**Sex**								
Male	30,192	49.7	2346	42.3	424	45.1	1291	41.3
Female	31,691	51.1	3153	57.7	495	54.9	1821	58.7
**Race**								
White, non‐Hispanic	48,959	68.7	4873	79.8	793	76.7	2772	79.5
Black, non‐Hispanic	4807	12.6	304	10.5	64	14.6	167	11.2
Asian, non‐Hispanic	1331	3.7	22	1.4	4	0.7	13	1.5
American Indian/Alaskan Native	594	1.1	56	1.4	11	1.2	32	1.4
Hispanic/Latino	5015	10.7	161	4.2	34	4.4	86	3.9
Other, non‐Hispanic	1177	3.2	83	2.8	13	2.4	42	2.4
**Age group**								
18–49	25,194	54.1	540	15.3	91	15.2	330	15.1
50–64	18,073	26.7	1402	31.5	259	33.8	799	30.8
65+	18,113	19.3	3526	53.2	564	50.9	1963	54.1
**Urban/rural status**								
Urban	54,506	92.5	4742	92.1	806	90.1	2748	93.3
Rural	7377	7.5	757	7.9	113	9.9	364	6.7
**Housing tenure** ^d^								
Own	45,563	75.4	4565	87.0	764	87.4	2620	87.8
Rent	16,320	24.6	934	13.0	155	12.6	492	12.2
**Income levels**								
Less than $50,000	22,966	39.1	2407	44.9	409	47.9	1255	42.4
$50,000 to < $100,000	19,723	30.7	1774	30.7	281	28.2	1037	31.2
$100,000 or more	19,194	30.2	1318	24.4	229	23.9	820	26.4
**Education levels**								
Graduated from high school or less	17,326	36.4	1443	33.9	252	36.9	725	30.9
At least some college/technical school	44,557	63.6	4056	66.1	667	63.1	2387	69.1
**Employment status**								
Employed	37,093	65.0	1683	35.5	274	30.7	1041	37.5
Retired	16,023	17.9	3065	48.5	467	45.6	1721	49.3
Not working^e^	8767	17.0	751	16.0	178	23.7	350	13.2

^a^ This sample excludes participants with any missing data for the housing outcomes, cancer diagnosis history, cancer types, sex, race, age, education levels, urban status, housing tenure, income, and employment status.

^b^ Excluding all skin cancers (melanoma and nonmelanoma).

^c^ Excluding participants with missing cancer treatment history and those who either refused treatment, had not started treatment, or reported treatment was unnecessary.

^d^ Excluding participants with an “other living arrangement” response.

^e^ Including those who were unemployed, those who were unable to work, homemakers, and students.

### 3.2 Prevalence of Housing and Utility Insecurity by Cancer History and Treatment Across Housing Tenure

**FIGURE 2:**
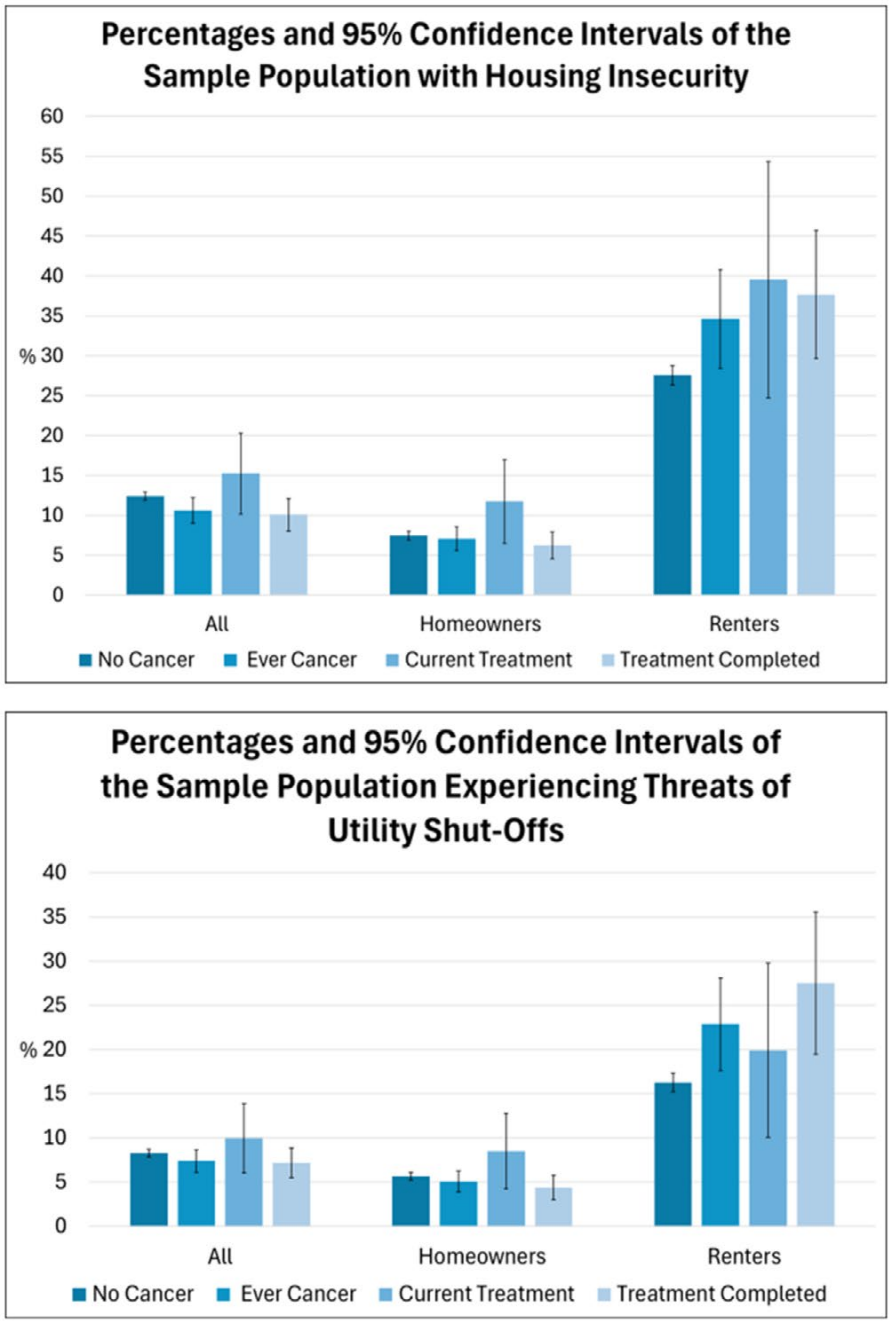
Percentages of the sample population with housing and utility insecurity by housing tenure. The sample excludes participants with missing data on housing outcomes, cancer history, cancer type, demographics, socioeconomic factors, or housing tenure (those reporting “other living arrangement” were excluded). “Ever Cancer” includes participants with a cancer diagnosis other than skin cancer (melanoma or nonmelanoma). “Current Treatment” and “Treatment Completed” are subgroups of “Ever Cancer,” limited to respondents with valid treatment data and excluding those who refused, had not started, or reported treatment was unnecessary.

Figure 2 (and Table S1) summarizes the prevalence of housing and utility insecurity by cancer diagnosis history, treatment history, and housing tenure. In general, individuals currently undergoing treatment reported a higher prevalence of housing and utility insecurity compared to individuals without a history of cancer and those who had completed treatment, both overall and after stratification by housing tenure. Throughout our results, however, clear differences were observed between homeowners and renters.

Overall, 15.24% (95% CI: 10.16–20.33) of those currently undergoing treatment and 10.08% (95% CI: 8.04–12.12) of those who completed treatment reported housing insecurity. Similar patterns were observed for utility insecurity, though these were lower in prevalence.

Renters, however, reported significantly higher prevalence of housing and utility insecurity across all groups (no cancer, ever cancer, current treatment, and treatment completed) than homeowners. 34.66% (95% CI: 28.47–40.85) of renters with a history of cancer diagnosis, for example, reported housing insecurity, compared to 7.06% (95% CI: 5.59–8.53) of their homeowner counterparts. For utility insecurity, the prevalences were 22.85% (95% CI: 17.62–28.08) and 5.06% (95% CI: 3.88–6.25) among renters and homeowners with a cancer history, respectively.

Renters currently undergoing cancer treatment also reported substantially higher prevalences of housing insecurity (39.54%; 95% CI: 24.71–54.36) and utility insecurity (19.91%; 95% CI: 10.03–29.78) than those with no history of cancer (27.56% for housing insecurity and 16.27% for utility insecurity). Prevalences of both housing and utility insecurity remained high even after renters completed treatment, at 37.68% for housing insecurity and 27.49% for utility insecurity.

In contrast, among homeowners, cancer‐associated housing and utility insecurity remained confined to those currently undergoing cancer treatment. Homeowners in cancer treatment reported prevalences of 11.74% (95% CI: 6.49–17.00) and 8.49% (95% CI: 4.22–12.75) for housing and utility insecurity, respectively. Homeowners who completed cancer treatment reported lower prevalences similar to those with no history of cancer.

### 3.3 Demographic Predictors of Insecurity Among Cancer Survivors

Table 2 summarizes results from logistic regression models that analyzed associations between demographic factors and housing and utility insecurity among individuals with a history of cancer diagnosis. We present results from both bivariate models and multivariate models. After adjustment, associations for renting (vs. homeowning), being female (vs. male), being aged 18–49 or 50–64 (vs. aged 65 or older), being Latino/Hispanic or Black, Non‐Hispanic, having a high school degree or less, earning less than $50,000 annually (vs. earning $100,000 or more), and not working (vs. being retired) were still statistically significant for both housing and utility insecurity outcomes. Identifying as American Indian/Alaskan Native was significantly associated with experiencing threats of utility shut‐offs but not housing insecurity. Earning $50,000 to < $100,000 (vs. earning $100,000 or more) was associated with higher odds of housing but not utility insecurity.

**TABLE 2:** Modeling the effects of demographic and socioeconomic factors on housing insecurity among cancer survivors^a^.

	Housing insecurity		Threats of utility shut‐offs	
	OR (95% CI)	AOR (95% CI)	OR (95% CI)	AOR (95% CI)
**Sex**				
Male	Ref	Ref	Ref	Ref
Female	2.44* (1.66, 3.58)	1.99** (1.33, 2.98)	2.00* (1.35, 2.96)	1.61** (1.08, 2.41)
**Age groups**				
Age 65+	Ref	Ref	Ref	Ref
Age 50–64	3.28* (2.13, 5.05)	3.08** (2.00, 4.74)	2.34* (1.46, 3.75)	2.18** (1.37, 3.45)
Age 18–49	8.11* (5.14, 12.78)	6.61** (4.13, 10.58)	6.64* (4.01, 10.98)	5.47** (3.24, 9.25)
**Race/ethnicity**				
White, non‐Hispanic	Ref	Ref	Ref	Ref
Black, non‐Hispanic	2.39* (1.41, 4.04)	2.56** (1.49, 4.39)	2.16* (1.22, 3.83)	2.20** (1.20, 4.03)
Latino, Hispanic	4.04* (2.20, 7.44)	2.80** (1.50, 5.21)	4.26* (2.16, 8.41)	2.97** (1.42, 6.22)
American Indian/Alaskan Native	2.59 (1.01, 6.63)	2.79 (0.73, 10.66)	3.84* (1.56, 9.44)	4.19** (1.20, 14.63)
Asian, non‐Hispanic	1.18 (0.15, 9.18)	0.78 (0.08, 8.08)	0.26 (0.05, 1.39)	0.18 (0.03, 1.16)
Other, non‐Hispanic	1.90 (0.84, 4.30)	1.25 (0.52, 3.01)	2.05 (0.88, 4.77)	1.41 (0.58, 3.40)
**Education levels**				
At least some college	Ref	Ref	Ref	Ref
High school or less	2.09* (1.48, 2.95)	2.40** (1.67, 3.45)	1.93* (1.33, 2.81)	2.11** (1.42, 3.13)
**Housing tenure status** ^b^				
Homeowners	Ref	Ref	Ref	Ref
Renters	6.98* (4.89, 9.98)	2.06** (1.33, 3.20)	5.55* (3.78, 8.15)	1.71** (1.04, 2.83)
**Annual income levels**				
$100,000 or more	Ref	Ref	Ref	Ref
$50,000 to < $100,000	2.59* (1.23, 5.45)	3.92** (1.83, 8.41)	1.33 (0.60, 2.95)	1.81 (0.82, 4.02)
< $50,000	23.42* (12.79, 42.89)	30.58** (15.58, 59.99)	10.00* (5.07, 19.72)	10.15** (4.86, 21.22)
**Employment**				
Retired	Ref	Ref	Ref	Ref
Employed^c^	2.80* (1.74, 4.51)	1.36 (0.75, 2.46)	2.08* (1.22, 3.54)	1.04 (0.57, 1.91)
Not working^d^	10.94* (6.79, 17.64)	4.66** (2.60, 8.35)	7.87* (4.67, 13.26)	3.51** (1.89, 6.49)

Abbreviations: AOR, adjusted odds ratio; CI, confidence interval; OR, odds ratio; Ref, reference.

^a^ This sample includes participants who reported a history of cancer diagnosis other than skin cancer. We excluded all participants with skin cancers (melanoma and nonmelanoma) and those with any missing data for the housing outcomes, cancer types, sex, race, age, education levels, urban status, housing tenure, income, and employment status.

^b^ Excluding participants with an “other living arrangement” response.

^c^ Including participants who reported being employed for wages or self‐employed.

^d^ Including those who were unemployed, those who were unable to work, homemakers, and students.

* Statistically significant difference with false discovery rate correction (corrected *p* value ≤ 0.05).

** Statistically significant difference after adjustment for potential confounders. For sex, adjusted for age and race; for age, adjusted for sex and race; for race, adjusted for sex and age; for education, adjusted for sex, age, and race; for housing tenure, adjusted for sex, age, race, education, income, and employment; for income, adjusted for sex, age, race, education, and employment; for employment, adjusted for sex, age, race, and education.

### 3.4 Regression Models

Finally, Table 3 summarizes results from logistic regressions modeling associations between a history of cancer diagnosis or treatment and housing and utility insecurity, after adjusting for potential confounders, including sex, age, race/ethnicity, education, urban status, state, and interview month.

**TABLE 3:** Logistic regression models between cancer history/treatment and housing insecurity adjusted for state and interview month fixed effects, sex, age, education, urban status, and race^a^.

	No cancer	Ever cancer^c^			Current treatment^d^			Treatment completed^d^		
		AOR	95% CI		AOR	95% CI		AOR	95% CI	
**All** ^b^	Ref									
Housing insecurity		1.43*	1.18	1.74	1.96*	1.28	3.01	1.38*	1.09	1.76
Threats of utility shut‐offs		1.36*	1.09	1.69	1.67*	1.06	2.61	1.34*	1.02	1.78
**Homeowners**										
Housing insecurity		1.35*	1.05	1.75	2.16*	1.29	3.62	1.20	0.88	1.65
Threats of utility shut‐offs		1.24	0.93	1.65	1.93*	1.13	3.32	1.07	0.74	1.55
**Renters**										
Housing insecurity		1.87*	1.37	2.55	2.09	0.97	4.49	2.12*	1.47	3.06
Threats of utility shut‐offs		1.82*	1.28	2.57	1.39	0.69	2.80	2.30*	1.49	3.56

Abbreviations: AOR, adjusted odds ratio; CI, confidence interval; OR, odds ratio; Ref, reference.

^a^ Sex, age, education, urban status, and race are potential confounders adjusted in these models. We also adjusted for state and interview month fixed effects.

^b^ Including both renters and homeowners and excluding participants with an “other living arrangement” response to the housing tenure question.

^c^ Excluding all skin cancers (melanoma and nonmelanoma).

^d^ Excluding participants with missing cancer treatment history and those who either refused treatment, had not started treatment, or reported that treatment was unnecessary.

* Statistically significant difference.

Overall, a history of cancer diagnosis was associated with higher odds of reporting housing insecurity (AOR 1.43, 95% CI: 1.18–1.74) and utility insecurity (AOR 1.36; 95% CI: 1.09–1.69). Compared to those without a history of cancer diagnosis, patients currently undergoing treatment and those who completed treatment were also more likely to experience both housing insecurity (AOR 1.96; 95% CI: 1.28–3.01 and AOR 1.38; 95% CI: 1.09–1.76, respectively) and utility insecurity (AOR 1.67; 95% CI: 1.06–2.61 and AOR 1.34; 95% CI: 1.02–1.78). Renters who had a history of cancer diagnosis had significantly higher odds of reporting both housing and utility insecurity than renters without a cancer history (AOR 1.87; 95% CI: 1.37–2.55 and AOR 1.82; 95% CI: 1.28–2.57, respectively). Homeowners who had a history of cancer diagnosis were more likely to report housing insecurity than homeowners without a cancer history (AOR 1.35; 95% CI: 1.05–1.75); however, the adjusted odd ratio for utility insecurity among homeowners was not statistically significant (AOR 1.24; 95% CI: 0.93–1.65).

In models that considered treatment status and homeownership, results were generally consistent with patterns observed in Table 2. Among renters, those who completed treatment were significantly more likely to report both housing insecurity (AOR 2.12; 95% CI: 1.47–3.06) and threats of utility shut‐offs (AOR 2.30; 95% CI: 1.49–3.56). Renters who are currently undergoing treatment may also experience a higher odds of housing insecurity, although this estimate was not statistically significant due to the small sample size (AOR 2.09; 95% CI: 0.97–4.49). Among homeowners, compared to those without a cancer history, the odds of reporting housing and utility insecurity were significantly higher in the current treatment group (AOR 2.16; 95% CI: 1.29–3.62 and AOR 1.93; 95% CI: 1.13–3.32) but not statistically significantly different in the treatment completed group (AOR 1.20; 95% CI: 0.88–1.65 and AOR 1.07; 95% CI: 0.74–1.55).

Additionally adjusting for marital status and long COVID did not meaningfully alter our findings (Table S2).

## 4 Discussion

Our study explored three key questions to deepen understanding of housing and utility insecurity across the cancer care continuum. We first examined the prevalence of these outcomes among individuals currently undergoing cancer treatment and those who completed treatment. We then assessed whether a history of cancer diagnosis was associated with an increased risk. Lastly, we evaluated whether housing tenure and socioeconomic characteristics shape these associations.

We found that 10.64% and 7.37% of individuals with a cancer history reported housing and utility insecurity, respectively, figures slightly higher than those reported by Hacker et al. [24]. These differences may be attributed to our stricter inclusion criteria, including the exclusion of all skin cancers (melanoma and nonmelanoma), which are typically associated with better prognoses and lower financial burden [30].

Importantly, while the overall prevalence of housing and utility insecurity was lower among individuals with a cancer history compared to those without, individuals currently undergoing treatment experienced notably higher levels of both forms of insecurity. These findings underscore the financial toll that active cancer treatment imposes. Renters, in particular, face disproportionately high prevalences of both housing and utility insecurity across all groups. Among renters with a cancer history, the prevalence of housing insecurity reached 34.66%, and among those actively in treatment, it spiked to 39.54%, highlighting a vulnerable subgroup of cancer patients at heightened risk of material hardship.

Our findings further revealed that having a cancer diagnosis was significantly associated with increased odds of both housing and utility insecurity, even after adjusting for confounders. While previous studies [22, 24] did not find significant differences in housing insecurity between individuals with and without a cancer history, these inconsistencies may reflect differences in sample definitions, geographic scope, and the inability of prior studies to distinguish between individuals actively in treatment and those posttreatment. Our results suggest that these distinctions matter, both statistically and substantively.

When stratified by housing tenure, we observed nuanced but meaningful differences. Homeowners with a cancer history were more likely to report housing insecurity, but not utility insecurity, relative to homeowners without a cancer history. Among renters, however, a cancer diagnosis was associated with significantly higher odds of both housing and utility insecurity. Among homeowners, insecurity was more commonly reported during treatment than after treatment completion, which may reflect financial buffers such as home equity or foreclosure protections [31]. In contrast, renters who completed the treatment still exhibited a higher likelihood of both housing and utility insecurities.

These findings suggest a possible housing tenure survival bias, where homeowners who lost housing during treatment may have transitioned into rental housing and are thus no longer captured in the homeowner data. As a result, the homeowners who remain in the posttreatment group may be those financially stable enough to retain their housing through the course of care. While our data cannot directly test this hypothesis, the observed patterns warrant further exploration.

In multivariable analyses, we found that renting, female gender, younger age, being Latino/Hispanic or Black, Non‐Hispanic, completing high school or less, earning less than $50,000 annually, and not working (compared to being retired) remained significantly associated with both forms of insecurity. Identifying as American Indian/Alaskan Native was significantly associated with utility insecurity but not housing insecurity. These results are consistent with prior literature demonstrating the compounding impact of sociodemographic disadvantage on financial outcomes among cancer survivors [7, 10, 23].

Our findings underscore the urgent need for multilevel interventions that address housing‐related hardship among cancer patients and survivors, particularly renters and individuals with lower socioeconomic status. Cancer can destabilize employment, deplete financial resources, and exacerbate housing precarity, especially for those without the safety nets available to homeowners. Policies that provide financial assistance, rent and mortgage relief, employment protections, and anti‐eviction safeguards are essential, not only for protecting the financial health of cancer patients but also for improving their survival [32]. Programs such as Medicaid housing waivers, navigator‐led financial screenings, and hospital‐based social work services can play a key role in early identification and support for those at risk [33, 34, 35]. At the state level, programs such as New Jersey's state‐funded rental assistance, and strong utility shutoff protections in states like Mississippi and Idaho, may help reduce housing‐related hardship [36, 37]. Integrating housing support into oncology care could mitigate the long‐term consequences of financial hardship and promote more equitable outcomes for patients.

### 4.1 Study Strengths and Limitations

Several limitations should be noted. First, the BRFSS is a telephone‐based survey that excludes institutionalized populations and may be subject to recall or response biases, even with the use of sampling weights. Cancer history and treatment status are self‐reported and not verified through medical records. Our analytic sample was also restricted to data from 14 states, limiting the generalizability of the findings to the broader US population. Additionally, we were unable to account for residential history, such as duration of residence or frequency of moves, due to data limitations in BRFSS. This constrained our ability to fully capture housing stability within heterogeneous groups of renters and homeowners. Approximately 26.7% of cancer survivors (excluding those with skin cancer) did not report treatment status, and missingness was not entirely random. The nonresponse group differed slightly by race, education, and rural residence, which may introduce bias toward the null. Finally, this study is cross‐sectional and cannot establish causality. It remains unclear whether cancer increases the risk of housing insecurity due to treatment‐related disruptions and costs, or whether individuals with preexisting housing instability face increased cancer risk or worse outcomes (or both). Longitudinal studies are needed to examine the directionality and mechanisms underlying these associations.

This study also has important strengths. First, we extended previous analyses that used BRFSS data by leveraging both the Cancer Survivorship and the Social Determinants and Health Equity optional modules from the 2022 and 2023 BRFSS surveys. This dual‐module approach allowed for more nuanced subgroup analyses and enhanced our ability to distinguish between individuals currently undergoing cancer treatment and those who had completed treatment, an important differentiation. By excluding cases of melanoma and nonmelanoma skin cancer, we also reduced the likelihood of overestimating positive housing and financial outcomes often associated with cancers that carry a lower treatment burden and better prognosis. Furthermore, we evaluated the role of housing tenure and key sociodemographic characteristics, addressing critical gaps in the literature.

Most importantly, this study identified prevalences of housing insecurity among cancer survivors that warrant immediate action. The fact that nearly 2 in 5 renters and more than 1 in 9 homeowners undergoing cancer treatment are unable to pay their housing and/or utility bills means that housing insecurity may play an important role in population‐level cancer outcomes. Researchers, policymakers, and health systems must move quickly and decisively to prevent these patterns and safeguard cancer survivors' well‐being.

## Author Contributions

**Tina Duong Nguyen:** data curation (lead), formal analysis (lead), methodology (equal), project administration (equal), visualization (equal), writing – original draft (lead). **Jan M. Eberth:** conceptualization (supporting), funding acquisition (lead), project administration (equal), resources (equal), supervision (supporting), writing – review and editing (equal). **Elochukwu Ezenwankwo:** visualization (supporting), writing – review and editing (equal). **Gabriel L. Schwartz:** conceptualization (lead), methodology (equal), project administration (equal), supervision (lead), visualization (equal), writing – review and editing (equal).

## Funding

This work was supported by the National Institutes of Health (U54CA267735).

## Conflicts of Interest

The authors declare no conflicts of interest.

## Supporting information


**Tables S1–S2:** cam471436‐sup‐0001‐TablesS1‐S2.docx.

## Data Availability

The data that support the findings of this study are available in BRFSS at https://www.cdc.gov/brfss/index.html.
