# Supplementary material for: Housing Insecurity and Threats of Utility Shut‐Offs Among Cancer Survivors in the United States, BRFSS 2022–2023
Source: Cancer Med. 2025 Dec 4;14(23):e71436. doi: 10.1002/cam4.71436 (PMC12676617; doi:10.1002/cam4.71436)
Supplement: Supplementary file 1 — Tables S1–S2: cam471436‐sup‐0001‐TablesS1‐S2.docx. [file CAM4-14-e71436-s001.docx]

|  | **No Cancer** | | | **Ever Cancer^1^** | | | **Current Treatment^2^** | | | **Treatment Completed^2^** | | |
| --- | --- | --- | --- | --- | --- | --- | --- | --- | --- | --- | --- | --- |
|  | % | 95% CI | | % | 95% CI | | % | 95% CI | | % | 95% CI | |
| **All** |  |  |  |  |  |  |  |  |  |  |  |  |
| Housing Insecurity | 12.38 | 11.87 | 12.89 | 10.64 | 9.03 | 12.26 | 15.24 | 10.16 | 20.33 | 10.08 | 8.04 | 12.12 |
| Threats of Utility Shut-Offs | 8.26 | 7.82 | 8.69 | 7.37 | 6.10 | 8.65 | 9.92 | 6.01 | 13.83 | 7.19 | 5.51 | 8.87 |
| **Homeowners** |  |  |  |  |  |  |  |  |  |  |  |  |
| Housing Insecurity | 7.44 | 6.91 | 7.96 | 7.06 | 5.59 | 8.53 | 11.74 | 6.49 | 17.00 | 6.24 | 4.56 | 7.93 |
| Threats of Utility Shut-Offs | 5.64 | 5.19 | 6.10 | 5.06 | 3.88 | 6.25 | 8.49 | 4.22 | 12.75 | 4.37 | 2.98 | 5.76 |
| **Renters** |  |  |  |  |  |  |  |  |  |  |  |  |
| Housing Insecurity | 27.56 | 26.32 | 28.81 | 34.66 | 28.47 | 40.85 | 39.54 | 24.71 | 54.36 | 37.68 | 29.02 | 46.34 |
| Threats of Utility Shut-Offs | 16.27 | 15.21 | 17.34 | 22.85 | 17.62 | 28.08 | 19.91 | 10.03 | 29.78 | 27.49 | 19.47 | 35.52 |

Supplemental Materials: Housing Insecurity and Threats of Utility Shut-Offs
Among Cancer Survivors in the U.S., BRFSS 2022-2023

**Table S1:** **Percentages of the Sample Population* With Housing and Utility Insecurity by Housing Tenure****

*CI = confidence interval*

**The sample excludes participants with any missing data for the housing outcomes, cancer diagnosis history, cancer types, sex, race, age, education levels, urban status, housing tenure, income, and employment status*

***Excluding participants with an “other living arrangement” response to the housing tenure question*

*^1^Excluding all skin cancers, including both melanoma and non-melanoma*

*^2^Excluding participants with missing cancer treatment history and those who either refused treatment, hadn’t started treatment, or reported treatment was unnecessary​*

**Table S2: Logistic Regression Adjusted for State and Interview Month Fixed Effects, Sex, Age, Education, Urban Status, Race, Marital Status and Long COVID^1^**

|  | **No Cancer** | **Ever Cancer^3^** | | | **Current Treatment^4^** | | | **Treatment Completed^4^** | | |
| --- | --- | --- | --- | --- | --- | --- | --- | --- | --- | --- |
|  |  | AOR | 95% CI | | AOR | 95% CI | | AOR | 95% CI | |
| **All^2^** | ref |  |  |  |  |  |  |  |  |  |
| Housing Insecurity |  | 1.39* | 1.14 | 1.69 | 1.95* | 1.25 | 3.03 | 1.33* | 1.05 | 1.70 |
| Threats of Utility Shut-Offs |  | 1.31* | 1.05 | 1.64 | 1.65* | 1.04 | 2.59 | 1.30 | 0.98 | 1.72 |
| **Homeowners** |  |  |  |  |  |  |  |  |  |  |
| Housing Insecurity |  | 1.33* | 1.02 | 1.73 | 0.72 | 0.34 | 1.52 | 1.18 | 0.85 | 1.62 |
| Threats of Utility Shut-Offs |  | 1.21 | 0.91 | 1.62 | 1.94* | 1.13 | 3.31 | 1.04 | 0.72 | 1.51 |
| **Renters** |  |  |  |  |  |  |  |  |  |  |
| Housing Insecurity |  | 1.74* | 1.27 | 2.39 | 1.88 | 0.88 | 4.05 | 1.98* | 1.35 | 2.90 |
| Threats of Utility Shut-Offs |  | 1.72* | 1.22 | 2.44 | 1.21 | 0.59 | 2.48 | 2.23* | 1.45 | 3.43 |

*CI = confidence interval; OR = odds ratio; AOR = adjusted odds ratio; Ref = reference*

**Statistically significant difference*

*^1^Sex, age, education, urban status, race, marital status and Long COVID are potential confounders adjusted in these models. We also adjusted for state and interview month fixed effects.*

*^2^Including both renters and homeowners and excluding participants with an "other living arrangement" response to the housing tenure question*

*^3^Excluding all skin cancers (melanoma and non-melanoma)*

*^4^Excluding participants with missing cancer treatment history and those who either refused treatment, hadn’t started treatment, or reported treatment was unnecessary*
